# Inducting family physicians to offer primary care in remote areas of India is neither feasible nor necessary

**DOI:** 10.1016/j.lansea.2023.100246

**Published:** 2023-07-11

**Authors:** Nachiket Mor

**Affiliations:** Banyan Academy of Leadership in Mental Health, Thiruvidanthai, Kanchipuram, Tamil Nadu 603112, India

In their thoughtful piece, Ugargol and colleagues^1^ make a strong case for establishing a public-health cadre and the induction of “15,000 family medicine practitioners per year by the year 2030” to address the gaps in primary care. There is no debate on the importance of public health and primary care. However, for several reasons, it is unclear if the solutions they recommend are indeed the ones that hold the answers we seek:

1. Indian states are at very different stages in the evolution of their health systems, with, for example, many already having an adequate supply of primary care providers but significant gaps on the secondary care front (Table 1). There is also clear evidence that improvements in primary care can no longer drive further reductions in maternal and child mortality and that serious engagement with secondary care is needed.^3^^,^^4^

**Table 1:** The states are at very different stages.

	Full UHC states	Moderate UHC states	Basic healthcare states
	GHE > ₹2000	₹1000 > GHE > ₹2000	₹1000 > GHE
Adequate privately-provided comprehensive primary care	√	√	√
Adequate government-provided comprehensive primary care	√	x	x
Adequate government-provided secondary care	√	x	x
Adequate privately-provided secondary care	√	x	x
Adequate government-provided essential public health services	x	x	x
Adequate government-provided tertiary care	x	x	x
Adequate privately-provided tertiary care	√	√	√

UHC, universal health coverage; GHE, government health expenditure per capita at 2018 prices.

The classification into full UHC, moderate UHC, and basic healthcare has been drawn from Mor and Shukla.^2^
2. Government providers have struggled with delivering quality in primary care, driven principally by “know-do” gaps and not a lack of advanced training in family medicine.^5^
3. Ugargol and colleagues^1^ mistakenly take the view that a family physician at the level of the Primary Care Centre (PHC) can be the “first point of contact providing preventive, curative and rehabilitative services to the patient and his/her family” for 30,000 individuals when the British General Practitioner is able to manage only about a cohort of 2000 patients. The 2000-patient level within the government-owned health system is not the PHC but the sub-centre. Given the reality that it has proved nearly impossible to find trained medical practitioners to staff even district hospitals, and the rising demand for physicians worldwide, it is not clear if the required number of family physicians willing to work in government sub-centres can ever be found.

It is possible, however, that finding so many family physicians may not even be necessary. As discussed in Mor and colleagues,^6^ a physician-led, passive, acute-care-centred approach does not provide the kind of proactive, field-based, community-oriented, and culturally informed care that is necessary. Working with carefully defined protocols and modern technologies, Iran and the Indian Health System in Alaska have successfully demonstrated that the next generation of community health workers can do this far more competently and cost-effectively.^6^ Brazil, France, and Nigeria have effectively used existing pharmacies to provide care for chronic diseases.^7^ There is also the concern that family physicians relying as they do on their memories and the training that they have received, sometimes decades earlier, are prone to serious errors of judgement when dealing with conditions such as cancer, mental illness, COVID-19, and appropriate use of antibiotics.^6^

Instead of revisiting these older ideas of public health cadres and family physicians, the need may now instead be for a proactive approach which brings sensitivity to local cultures, social determinants, and essential public health functions to the forefront within a new and integrated vision of primary care as is being explored by organisations like Oak Street Health^8^ and El Centro Family Health.^9^ While a specialised public health cadre may still be helpful within such an approach in India, the family physician may not be a practically possible solution in all contexts.

## Contributors

NM is the sole author of this paper.

## Declaration of interests

The author reports no conflict of interest.
